# Force-based learning curve tracking in fundamental laparoscopic skills training

**DOI:** 10.1007/s00464-018-6090-7

**Published:** 2018-02-08

**Authors:** Sem F. Hardon, Tim Horeman, H. Jaap Bonjer, W. J. H. Jeroen Meijerink

**Affiliations:** 1 Department of Surgery, VU University Medical Center, P.O. Box 7057, 1007MB Amsterdam, The Netherlands; 2 Department of BioMechanical Engineering, Delft University of Technology, Delft, The Netherlands; 3 Department of Orthopedic Surgery, Academic Medical Center, Amsterdam, The Netherlands; 4 Department of Operation Rooms and MITeC Technology Center, Radboud University Medical Center, Nijmegen, The Netherlands

**Keywords:** Laparoscopic training, Force measurement, Tissue handling, Learning curve, Box trainer, Objective assessment

## Abstract

**Background:**

Within minimally invasive surgery (MIS), structural implementation of courses and structured assessment of skills are challenged by availability of trainers, time, and money. We aimed to establish and validate an objective measurement tool for preclinical skills acquisition in a basic laparoscopic at-home training program.

**Methods:**

A mobile laparoscopic simulator was equipped with a state-of-the-art force, motion, and time tracking system (ForceSense, MediShield B.V., Delft, the Netherlands). These performance parameters respectively representing tissue manipulation and instrument handling were continuously tracked during every trial. Proficiency levels were set by clinical experts for six different training tasks. Resident’s acquisition and development of fundamental skills were evaluated by comparing pre- and post-course assessment measurements and OSATS forms. A questionnaire was distributed to determine face and content validity.

**Results:**

Out of 1842 captured attempts by novices, 1594 successful trials were evaluated. A decrease in maximum exerted absolute force was shown in comparison of four training tasks (*p* ≤ 0.023). Three of the six comparisons also showed lower mean forces during tissue manipulation (*p* ≤ 0.024). Lower instrument handling outcomes (i.e., time and motion parameters) were observed in five tasks (resp. (*p* ≤ 0.019) and (*p* ≤ 0.025)). Simultaneously, all OSATS scores increased (*p* ≤ 0.028). Proficiency levels for all tasks can be reached in 2 weeks of at home training.

**Conclusions:**

Monitoring force, motion, and time parameters during training showed to be effective in determining acquisition and development of basic laparoscopic tissue manipulation and instrument handling skills. Therefore, we were able to gain insight into the amount of training needed to reach certain levels of competence. Skills improved after sufficient amount of training at home. Questionnaire outcomes indicated that skills and self-confidence improved and that this training should therefore be part of the regular residency training program.

Minimally invasive surgery (MIS) is increasingly the preferred surgical access in the operating theater. Therefore, simultaneously, the interest for training programs to teach technical skills is gaining ground rapidly. Trainees are expected to spend a minimum period of time in the surgical residency program, working on real patients under supervision, with the expectation that they will acquire all competences a qualified surgeons need [1–3]. Although this training model has been the standard since it was introduced by William S. Halsted in 1904, there are no objective data supporting the assumption that the number of years spent in residency is adequate [4, 5]. In fact, there is some evidence that the opposite might be true [1, 3]. Laparoscopic skills training is being challenged by different influences, such as the boundaries of the traditional apprentice-tutor model, the ethical objective to limit patient morbidity and error rate during surgery, and the continuous pressure on cost effectiveness [6, 7]. Simultaneously, time spent in the operation room (OR) is declining worldwide due to regulations that have reduced the legal number of working hours [8, 9]. Combined with these challenges, increased expectations of surgical outcomes necessitate to think about new design for a laparoscopic skills training curriculum for surgical residents [10]. Surgical education consists of acquiring knowledge, ability, skills, and performance. But how to assess all pillars? With a new design for a curriculum, comes the idea for different methods of assessment.

In 2007, a report by the Dutch Health Care Inspectorate (IGZ) concluded that actions to prevent complications in MIS were insufficient and that there was no uniform consensus on training in laparoscopic surgery [11]. Since then several initiatives have been proposed to improve training in MIS and to transfer learning curves out of the operating room [12]. Not only (bi)manual dexterity and hand-eye coordination, but also handling long instruments that amplify tremors, dealing with the fulcrum effect and reduced tactile feedback should be mastered to perform laparoscopic surgery safely [13, 14].

If laparoscopic experience is acquired in theater without preclinical training, it will have negative effects on learning curves and self-confidence will establish slowly, because residents are not comfortable due to a lack of competence [14]. Moreover, there is a higher risk for complications if the novice attains basic laparoscopic skills in a patient model [15]. These factors give occasion for an observational pilot study for a new preclinical curriculum for surgical residents.

Many factors affect the successful incorporation of simulator training into a surgical curriculum. One of the most important factors is trainee’s motivation [16]. In the case of surgical residents, fatigue, long working hours, limited free time, interference with clinical responsibilities, and operating room experience can all negatively affect a trainee’s motivation to participate in a skills curriculum [17]. This is why we executed a protocol, in which residents could train in their home situation without these external stressors, potentially compromising the effect of training.

As stated by ten Cate et al., the central focus of any innovation in postgraduate training should be supporting the individual supervisor in the daily practice of clinical teaching, not just to optimize the success of innovations in postgraduate training [18]. We aimed to establish a dynamic process that was tailored to individual needs and was continuously optimized based on accumulated evidence and experience [17]. By combining measurements of force and motion, fundamental laparoscopic skills in a box trainer model can be evaluated objectively and levels of competence can be determined more accurately [19–22]. It provides resident’s supervisor an objective tool to assess whether the trainee has reached the minimum required level of skill to actually start performing MIS in the OR [23].

The objective of this study was to gain insight into improvement of tissue manipulation and instrument handling skills, based on learning curves expressed in parameter outcomes. We aimed to show a decrease in parameter outcomes between pre- and post-course assessment for each of the six tasks for basic laparoscopic skills. Furthermore, in this study, we examined the adequate timespan (i.e., number of trials) to reach proficiency levels. Based on this information, we set standards for a newly developed at-home training curriculum.

## Materials and methods

### Systems and hardware

**Fig. 1:**
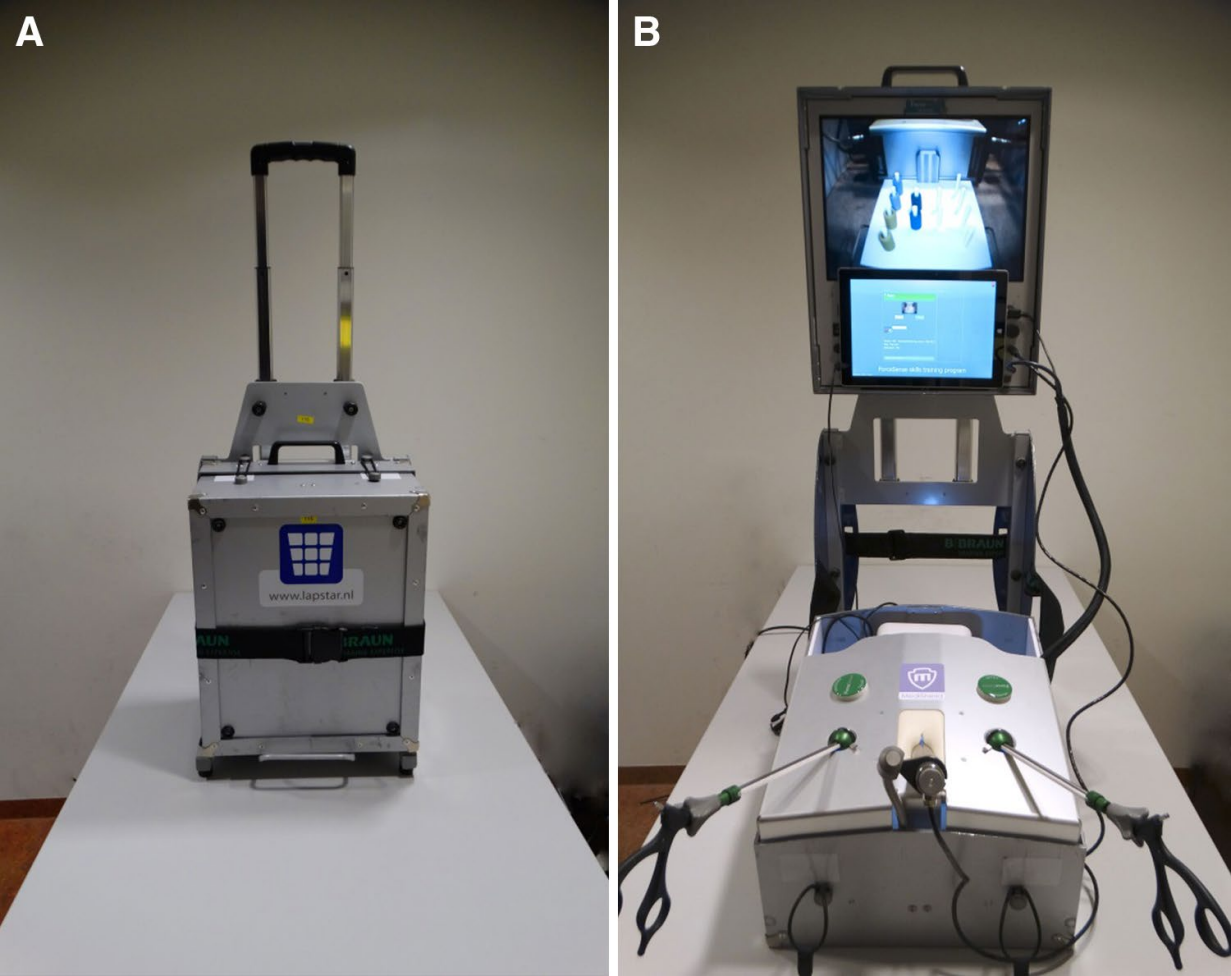
Box trainer equipped with the ForceSense system, measuring the tissue interaction forces and motion of instruments (**A** Box trainer packed for transport. **B** Box trainer installed for training.)

Two compact and portable laparoscopic simulators were used for this training program. For this research, we used box trainers measuring 45 × 30 × 25 cm as displayed in Fig. 1. The LAPSTAR training system (Camtronics B.V., Son, The Netherlands) was prepared to use the same instruments as used in the OR [24]. For this curriculum, trainees used two curved Maryland dissection forceps (B Braun Medical B.V., Melsungen, Germany).

A recently launched tracking, monitoring, and assessment system ‘ForceSense’ (MediShield B.V., Delft, The Netherlands) for box trainers provides world’s first force, motion, and time-based surgical assessment. Amongst many other parameters, it measures maximum absolute force, mean force during tissue manipulation, distances traveled by instrument tips, and time to complete the task. These parameters, as evaluated and described by Horeman et al., are considered representative for tissue manipulation and instrument handling skills. These parameters are displayed in Table 1 [20, 23].

**Table 1:** Description of objective performance metrics [23]

Parameter	Description
Task time	Task time (time needed to complete the task) is presented in seconds
Max absolute force	The highest absolute force (Newton) applied on the training task during the measurement was considered the max absolute force
Mean force during tissue manipulation	*i.e., Mean Force Non Zero* The force averaged across all samples during which force was exerted so that the resulting measure is based only on the periods of time when interaction/tissue manipulation took place. An example is given in Fig. 4, where the mean nonzero force would be calculated without forces measured during the periods in the red ovals. These circles represent the periods in which instruments do not manipulate tissue, skin pads, or tasks
Path Length (Left + Right Instrument)	The distances the left and right instrument tip traveled in a confined 3D space after completion of a training task were called path length left and path length right, respectively. The distance is presented in millimeters. The sum of path lengths of both instrument tips is presented as *Path Length Total*
Force penalties	A penalty was imposed if executed forces were above thresholds, as described in Table 2. Crossing the lower limit resulted in 1 penalty point. Crossing the upper limit, of time limit resulted in 10 penalty points

The laparoscopic box trainer is mobile, and thus, the trainee could set it up anywhere he or she prefers. At-home training was advised based on prior research [25]. All data from measurements and moments of training were directly logged in an online database, including a captured video of performance. An online interface enabled the possibility to receive presentations of results instantly.

### Assessment

**Fig. 2:**
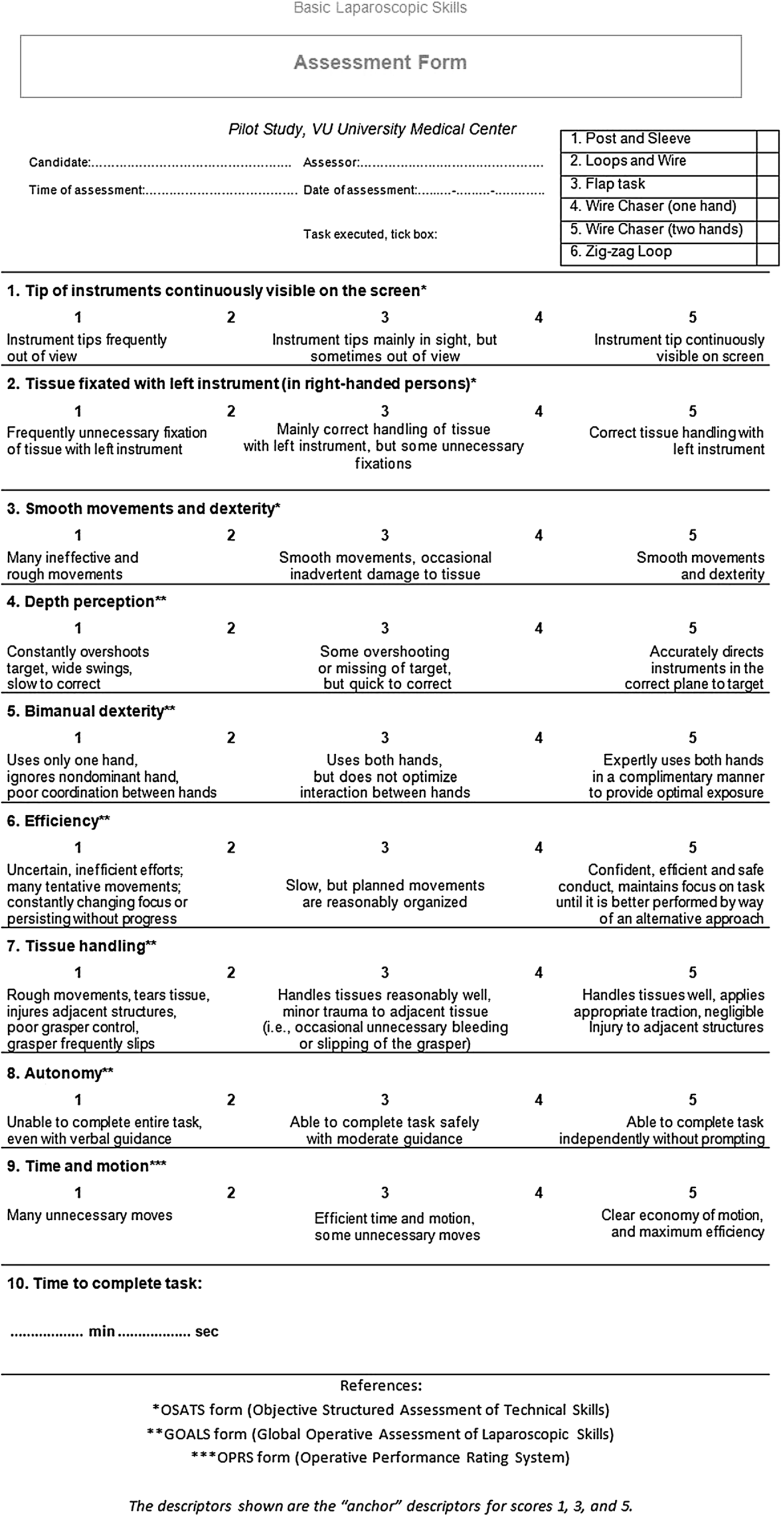
Assessment form (OSATS, GOALS, and OPRS combined)

**Fig. 3:**
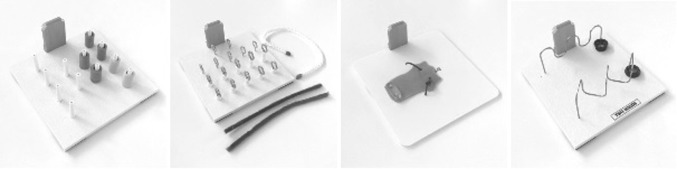
Task inserts for Basic Laparoscopic Skills training (see “Appendix” for detailed information)

In previous studies, feedback was consistently found to be a powerful method for improving surgical performance in terms of metrics, such as instrument movement [10]. By analyzing force metrics in addition to motion parameters, trainee’s competence can be determined more accurately. Parameters were selected based on their discriminating power and informative character [26]. Subjective assessment was analyzed by evaluation of a form (Fig. 2), derived from “Objective Structured Assessment of Technical Skills” (OSATS), “Global Operative Assessment of Laparoscopic Skills” (GOALS), and “Operative Performance Rating Scale” (OPRS) forms. Measurement data from validated laparoscopic tasks (Fig. 3) are very applicable to determine the level of competence during the training curriculum [13]. Both ForceSense metrics and OSATS outcomes were used for pre- and post-course assessment based on statistical analysis to identify learning effects. Participants were given a pre-course assessment at the beginning of the training curriculum and an post-course assessment at the end. During the pre- and post-course assessment, each of the six training tasks was measured.

**Table 2:** Proficiency levels per task, presented as mean ± standard deviation (SD)

Parameter	Task 1	Task 2	Task 3	Task 4	Task 5	Task 6
Time to complete task (s)	98.40 ± 23.78	76.58 ± 18.53	42.84 ± 10.26	41.11 ± 7.02	106.58 ± 16.25	56.45 ± 11.99
MaxForce (N)	1.72 ± 0.37	3.01 ± 0.82	1.56 ± 0.97	1.69 ± 0.67	1.22 ± 0.24	2.70 ± 0.50
MeanForceNZ (N)	0.45 ± 0.04	0.78 ± 0.13	0.49 ± 0.13	0.49 ± 0.11	0.43 ± 0.06	0.43 ± 0.06
Path Length total (mm)	4812.24 ± 805.64	3425.40 ± 590.56	1993.19 ± 612.29	1688.36 ± 457.69	4558.02 ± 353.39	3346.09 ± 974.55
Force penalties (#)	0	0	1	1	0	0

### Determining proficiency levels

The performances of seven expert surgeons were measured to determine proficiency levels. Table 2 shows parameter outcomes and standard deviation per parameter for each. The surgeons, affiliated with the VU University Medical Center (Amsterdam, The Netherlands), were selected based on prior experience (no. of advanced procedures *N* > 50) to determine the proficiency levels. Demographic characteristics for all surgeons are given in Table 3. Before each training task, the surgeon reads the short instructions, and watched the online video instructions of the executed tasks with a verbal explanation of each task. Then the surgeons executed six dissimilar tasks for basic laparoscopic skills. Parameter outcomes were recorded to determine proficiency levels for the resident’s training curriculum.

**Table 3:** Participant demographics

Demographics	Novices	Experts
Gender		
Male	3	5
Female	3	2
Hand dominance		
Right	4	7
Left	2	
Surgical (/medical) specialty		
General surgery	6	
GI surgery		7
Experience with laparoscopic box training (no. of times)		
None	3	2
1–5	3	1
6–10		1
11–20		
> 20		3
Experience with laparoscopic box training (hours)		
None	3	2
1–5	3	1
6–10		1
11–20		
21–50		1
> 50		2
Experience with laparoscopic virtual reality training (no. of times)		
None	3	4
1–5	3	1
6–10		
11–20		2
> 20		
Experience with laparoscopic virtual reality training (hours)		
None	3	4
1–5	3	1
6–10		
11–20		1
21–50		1
> 50		
Laparoscopic experience in theater (no. of advanced procedures^a^)		
None	4	
1–10	2	
10–50		
50–100		2
> 100		5

^a^Any other laparoscopic procedure than cholecystectomy, inguinal hernia repair, and/or appendectomy

The first run was used for familiarization with the equipment. The second run was measured and used to determine the expert level out of mean scores for maximum force (N), path length (mm), and time (s). During this performance, a video was captured from the laparoscope. Most illustrative videos of how to perform the training tasks were used for instructions for novices during the course.

### Feedback and penalties

**Fig. 4:**
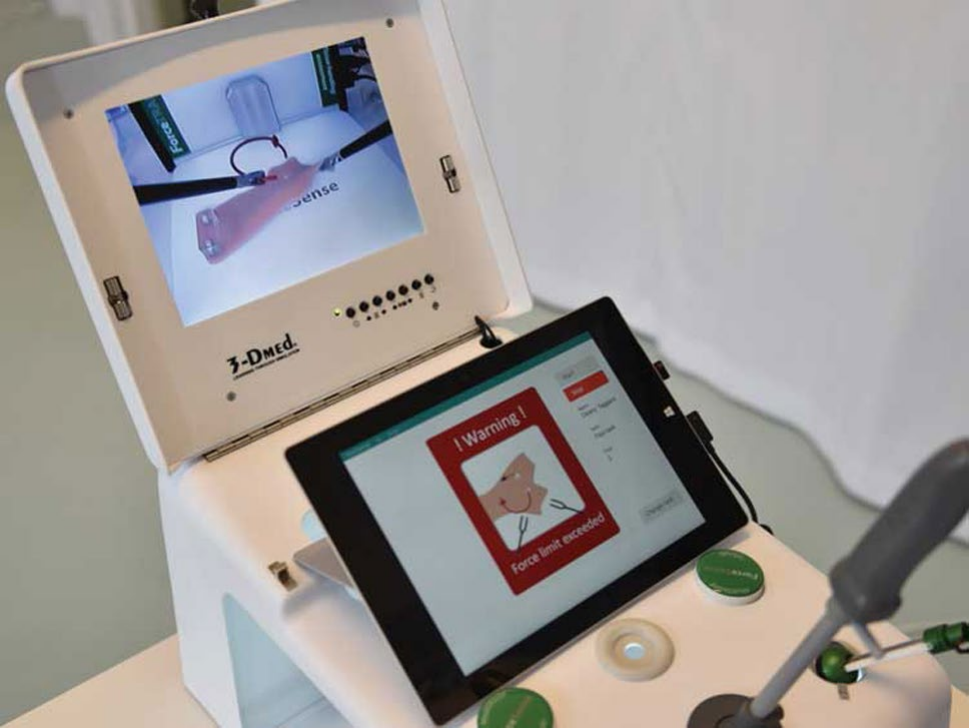
Instant force feedback system shown on tablet

**Fig. 5:**
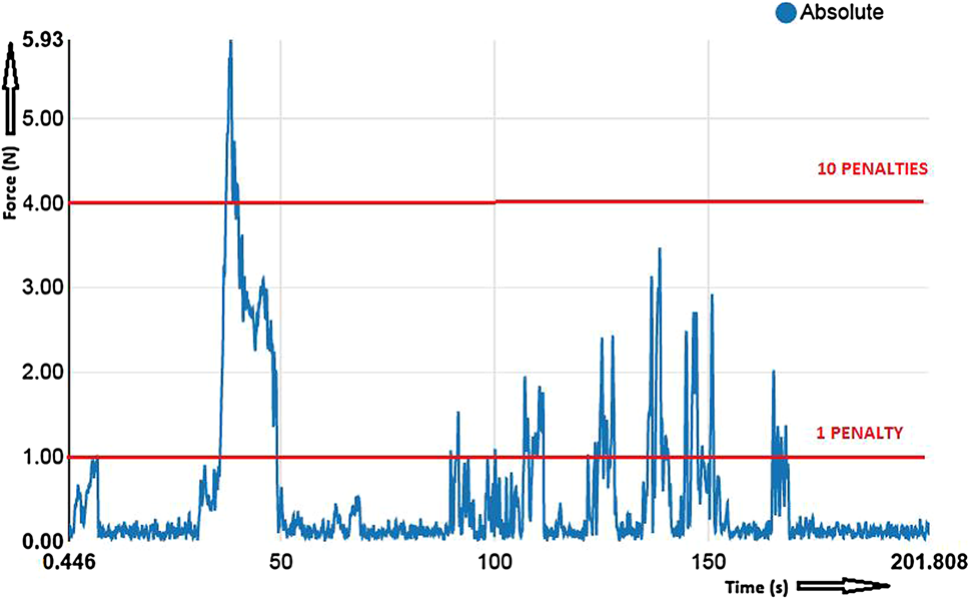
Force plot example, task 3 “Flap task”

During every performed trial, trainees received visual force feedback on the tablet connected to the ForceSense system (Fig. 4). The reference values for force penalties were set by MediShield B.V., Delft as determined in previous studies conducted with the ForceSense by Horeman et al. Thresholds for exceedance were set in absolute force metrics. Participant could receive 1 or 10 penalties for each excessive force peak. For task 1 “Post and Sleeve,” task 4 “Wire Chaser (one hand),” task 5 “Wire Chaser (two hands),” and task 6 “Zig-zag loop,” thresholds for 1 or 10 penalty points were, respectively, set at 2N and 4N. For task 2 “Loops and wire,” thresholds for 1 or 10 penalty points were, respectively, set at 4N and 8N. For these 5 tasks, a limit for 10 penalty points was set at 1 s of continuous excessive force. For task 3 “Flaptask,” thresholds for 1 and 10 penalty points were respectively set at 1N and 4N. Figure 5 shows an example of marked thresholds in an evaluated force plot.

### Participants

Secondly, we enrolled first year surgical residents with no prior or limited prior laparoscopic experience. A total of six novice surgical residents were included in this study. Demographic characteristics for novices are also displayed in Table 3. All trainees were employed at the Department of Surgery and they were all able to practice a minimum of 4 times per week and 15 min per training. Participants received online video-instructions. During the baseline performance the tasks, trainees were assessed by the assessment form by his or her own trainer in an affiliated clinic. Performances were simultaneously being measured by the ForceSense system. The baseline-test was also used to determine innate abilities and initial skillset. Trainees then trained at home, where to perform the training tasks until proficiency levels were reached. They were instructed to train approximately 4–5 days per week with a minimum of 15 min per training.

### Training tasks

The protocol contained five tasks created out of four platforms for clinical skill development in MIS (3-Dmed®, Franklin, OH, USA). Each task had different objectives as described in Fig. 3 and the Appendix [13]. For example hand-eye coordination, bimanual dexterity, depth perception, and interaction of the dominant and non-dominant hand. In addition a sixth task, the validated ‘Flap task,’ is added to this course for specific bimanual tissue handling manipulation (MediShield B.V., Delft, the Netherlands) [23].

### Statistical analyses

Data from ForceSense software and OSATS forms were analyzed by using SPSS Statistics 22.0 (SPSS Inc., Chicago, Illinois, USA). We used a paired sample *T*-test to analyze statistical differences between pre- and post-course assessment for each ForceSense parameter and OSATS. Differences were considered statistically significant if *p* < 0.05. A Pearson Correlation test was performed to detect if there was any correlation between the number of trials needed to reach proficiency for each parameter. Correlation was considered significant at the 0.01 level (2-tailed).

## Results

For the novice group, a total of 1842 performances were recorded, of which 1594 performances were executed successfully. We found significant differences between pre- and post-course assessment test for several parameters in every performed task. Table 4 summarizes changes in parameters measured by ForceSense presented as mean ± SD.

**Table 4:** Results of the novices, presented as mean ± standard deviation (SD)

Parameter	Training tasks	Pre-test	Post-test	*p* Value
MaxForce (N)	1. Post and Sleeve	2.31 ± 0.49	1.84 ± 0.34	0.127
	2. Loops and Wire	4.38 ± 1.61	2.02 ± 0.43	*0.023*
	3. Flap task	1.76 ± 0.80	0.84 ± 0.24	0.062
	4. Wire chaser	2.27 ± 0.87	1.15 ± 0.51	*0.022*
	5. Wire chaser bimanual	1.83 ± 0.73	1.03 ± 0.30	*0.013*
	6. Zig-zag loop	4.51 ± 1.49	2.40 ± 0.67	*0.007*
MeanForceNZ (N)	1. Post and Sleeve	0.48 ± 0.08	0.49 ± 0.09	0.842
	2. Loops and Wire	0.86 ± 0.15	0.65 ± 0.14	*0.021*
	3. Flap task	0.50 ± 0.11	0.36 ± 0.03	*0.024*
	4. Wire chaser	0.48 ± 0.03	0.42 ± 0.12	0.273
	5. Wire chaser bimanual	0.47 ± 0.11	0.50 ± 0.16	0.538
	6. Zig-zag loop	0.79 ± 0.13	0.65 ± 0.09	*0.011*
Time (s)	1. Post and Sleeve	204.09 ± 65.12	78.08 ± 14.22	*0.004*
	2. Loops and Wire	266.83 ± 159.19	48.56 ± 9.52	*0.019*
	3. Flap task	208.71 ± 184.02	41.91 ± 19.36	0.080
	4. Wire chaser	93.55 ± 30.84	20.49 ± 4.40	*0.003*
	5. Wire chaser bimanual	216.89 ± 54.81	65.07 ± 18.30	< *0.001*
	6. Zig-zag loop	188.34 ± 69.02	53.42 ± 15.55	*0.004*
Path Length (mm)	1. Post and Sleeve	9041.86 ± 2212.63	4781.04 ± 1030.44	*0.008*
	2. Loops and Wire	9949.71 ± 5642.17	2841.58 ± 181.54	*0.025*
	3. Flap task	8195.13 ± 7520.92	2114.45 ± 1043.19	0.116
	4. Wire chaser^a^	2624.20 ± 509.49	1141.92 ± 111.76	*0.001*
	5. Wire chaser bimanual	7610.43 ± 1253.56	3900.61 ± 728.38	*0.001*
	6. Zig-zag loop	7800.17 ± 2965.93	3107.90 ± 822.95	*0.011*
OSATS (mean score^b^)	1. Post and Sleeve	3.21 ± 0.39	4.56 ± 0.26	*0.001*
	2. Loops and Wire	2.86 ± 0.50	4.58 ± 0.27	*0.002*
	3. Flap task	2.88 ± 0.40	4.32 ± 0.58	*0.012*
	4. Wire chaser	3.28 ± 0.38	4.88 ± 0.14	< *0.001*
	5. Wire chaser bimanual	2.82 ± 0.40	4.11 ± 0.61	*0.028*
	6. Zig-zag loop	2.96 ± 0.39	4.37 ± 0.50	*0.005*

Significant *p* values are given in italics (*p* < 0.05)

^a^Path Length of dominant hand for task 4 “Wire Chaser (one hand)”

^b^Mean scores derived from 9 components of 5 point Likert scales

We found that on average it took the six participants at maximum 6.67 ± 4.97 (mean ± SD) trials to reach the proficiency level for Forces, 11.50 ± 7.15 (mean ± SD) for Path Length and 18.00 ± 12.39 (mean ± SD) for Time. These outcomes were counted for the sixth training task. The average number of trials to reach proficiency levels is presented in Table 5 for each task.

**Table 5:** Average number of trials needed to reach proficiency levels, presented as mean ± standard deviation (SD)

	Task 1	Task 2	Task 3	Task 4	Task 5	Task 6
MaxForce (N)	3.50 ± 2.43	2.17 ± 0.75	2.17 ± 1.60	2.17 ± 1.47	5.20 ± 2.68	6.67 ± 4.97
Path Length total (mm)	10.17 ± 9.54	15.33 ± 8.64	7.33 ± 4.37	5.83 ± 3.49	4.50 ± 1.76	11.50 ± 7.15
Time (s)	11.17 ± 11.13	11.17 ± 7.03	12.50 ± 9.87	4.00 ± 2.00	4.33 ± 2.94	18.00 ± 12.39

A significant correlation was seen in the Time versus Path Length comparison of the first task (*p* = 0.001). No further significant correlation was observed between comparison of Force versus Time, Time versus Path Length and Force versus Path Length for all six tasks.

For task 1 “Post and Sleeve,” there was a significant decreases between pre- and posttest for time (*p* < 0.01) and path length (*p* < 0.01). Same results were seen for task 4 and 5, respectively “Wire Chaser (one hand)” and “Wire Chaser (two hands).” Outcomes of task 2 “Loops and Wire” showed significant decreases for Time (*p* = 0.02), MaxForce (*p* = 0.02), MeanForceNZ (*p* = 0.02), and Path length (*p* = 0.03). Task 3 “Flaptask” metrics only showed decrease in MeanForceNZ (*p* = 0.02).

Most statistical significant decreases in parameter outcomes were demonstrated in task 6 “Zig-zag loop”; time (*p* < 0.01), MaxForce (*p* = 0.01), MeanForceNZ (*p* = 0.01), and path length (*p* = 0.01). Significant improvement of OSATS—scores was demonstrated for all tasks.

**Fig. 6:**
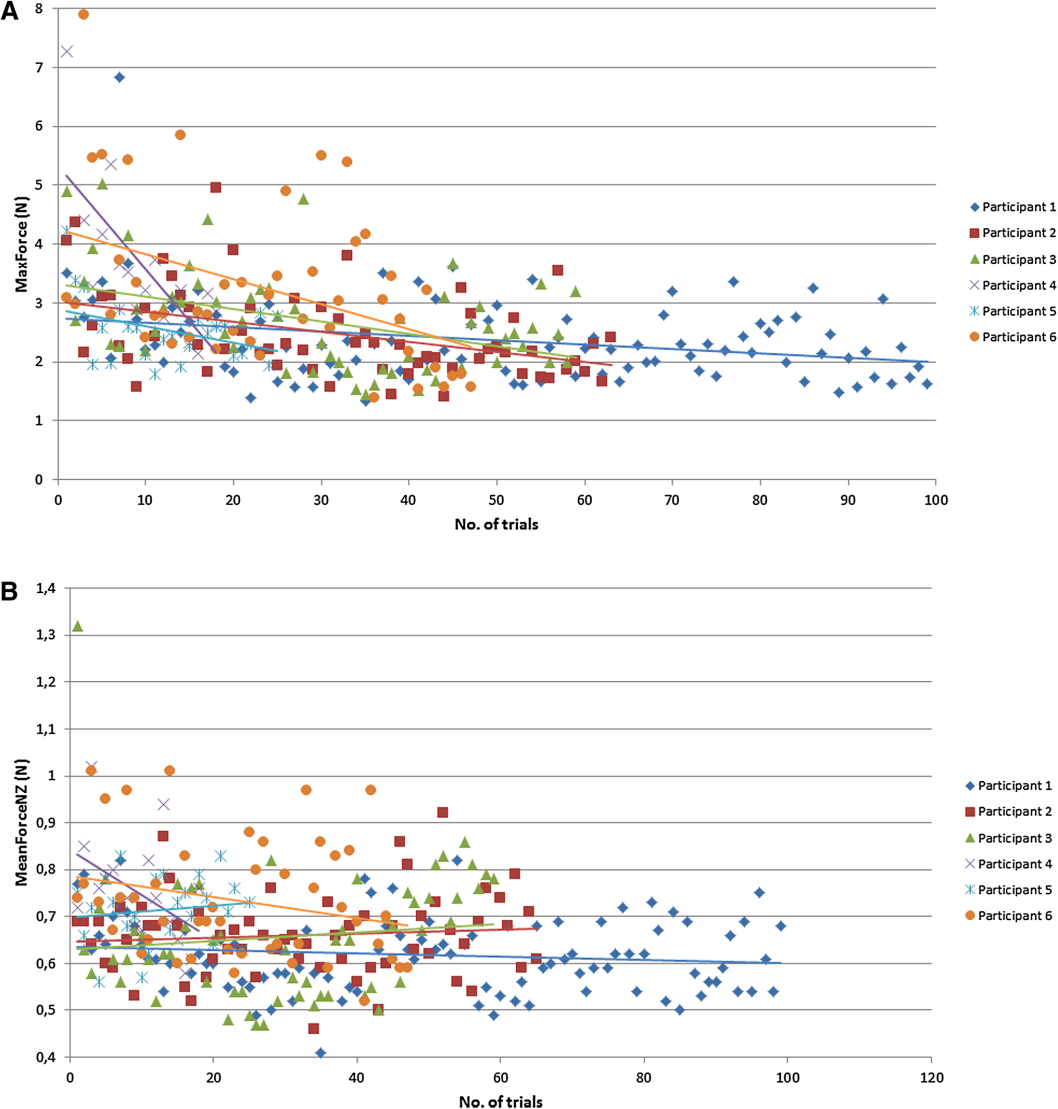

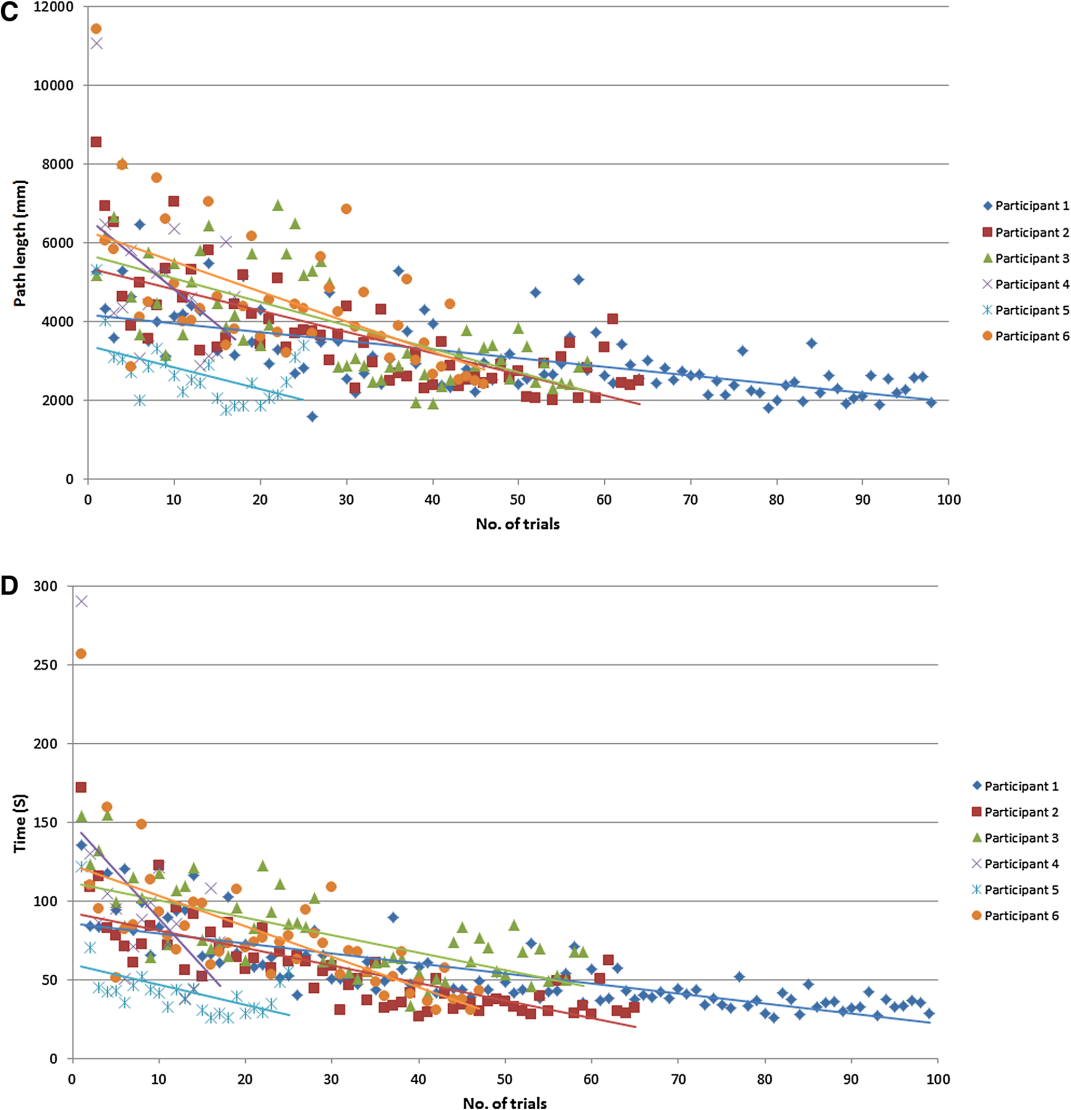
Force, motion, and time learning curve plots. **A** MaxForce (N). **B** MeanForceNZ (N). **C** Path Length (mm). **D** Time (s)

Based on results as shown in Table 4, task 6 was analyzed. A total of 367 performances were recorded among the six participants. Individual progress of one participant is presented for this task in Fig. 6A–D.

## Discussion

We analyzed changes in parameter outcomes and OSATS scores for each task to detect if the ForceSense metrics decreased significantly for different types of training tasks and proficiency levels were reached during the course. Within MIS, structural implementation of courses and structured assessment of skills are challenged by availability of trainers, time, and money. The results of this study show that the provided curriculum is effective for autonomous skills training, without compromising expensive working hours from both novices and trainers.

Different from the clear learning curves for the task time, path length and max force parameters, learning curves for the MeanForceNZ were less present for the six training tasks. This corresponds with the results from Horeman et al. [26], indicating that the MeanForceNZ parameter mainly represents one’s natural level of tissue interaction force, which can only been altered when applying active force feedback to the trainee.

The correlation results indicate that force, motion, and time parameters are not correlated. This is consistent with earlier results of Horeman et al. [23] and indicates that monitoring of time and motion parameters is not enough to predict tissue manipulation behavior. Therefore, it is advisable to first train surgeons specifically on efficient tissue manipulation and instrument handling before allowing them to focus on efficiency of time and complete the task as fast as possible. In order to pass the exam, the student should score beneath the threshold levels of all predefined parameters for that specific task. Table 5 shows that it takes a student on average between 11 and 36 trials to master a task based on force, motion, and time parameters. When the number of tasks is multiplied with the average time per task given in Table 2, we found that on average it takes the novices 146 min to reach proficiency levels for each task in this course. With an assumed attention span of 15 min and taking the standard deviations in mind, it is advisable to instruct novices to train a minimum of five times per week, for 2 weeks on the at home training system.

Principles of deliberate practice were applied by making use of goal-oriented training, objective performance metrics, and structural feedback. These factors, in addition to adequate assessment of skills and repetition of performances, contribute to effective and efficient acquisition of laparoscopic skills and a good start of any young surgeons career [17, 27, 28]. As previous studies indicated, skills are preferably acquired in a preclinical setting. After optimization of technical skills sets in a preclinical setting, attained skills can directly be transferred to the OR where they can endure [29, 30].

This prospective cohort pilot study was designed to evaluate possibilities for future training and research. We found that there are significant differences between in pre- and post-course outcomes of the motion and time parameters of task 1, 4, and 5, which are suitable for training in instrument handling. Task 2, 3, and 6 are more useful for training in tissue manipulation, and as a result we also found significant force parameter outcomes. Data of task 3 are in line with its training purpose, since it was specifically created to train bimanual tissue manipulation skills.

Major findings are significant changes between pre- and post-course assessment for every task, accurate determination of *MaxForce* and *Mean Force Non Zero* level improvements and insight in resident’s motivation and learning curves. We found that from all tasks, tasks 6 “Zig-zag loop” was most efficient for training fundamental skills, as it showed to be most discriminating for all parameters. This can be explained by its relatively strategic character, based on baseline test analysis and evaluation of captured video’s. Most advantage was attained by training this tasks.

Face and content validity was obtained by questionnaires at the end of the curriculum. Main findings are that skills and self-confidence improved during the course. Considering the answers given, we think this curriculum should be part of our regular surgical residency training program.

Improved preclinical learning curves and in-time training will result in individualization of surgical training for residents and shortening of surgical training [7]. If a resident finds positive results by tracking his or her learning curve, he or she gets motivated and will improve competences due to deliberate practice [27]. This will positively influence the self-confidence of residents in a preclinical setting as indicated by the results of the post-training questionnaires, displayed in Table 6. Face validity of box trainer and training tasks was also obtained from these surveys and presented in Tables 7 and 8. Based on the answers given, we concluded the protocol and materials appear effective.

**Table 6:** Content validity (general statements)

Statement	Visual Analog Scale (VAS) 0–100 mm Presented as mean ± SD (in mm)
Content protocol	
The box is valuable (/useful) for laparoscopic training	91.33 ± 10.78
How suitable are the tasks for acquisition of basic laparoscopic skills?	80.17 ± 14.52
How valuable are the ForceSense metrics in addition to the OSATS form?	80.67 ± 19.50
The curriculum should be part of the regular surgical resident training	94.33 ± 7.17
Timespan of the curriculum	78.67 ± 23.49
Duration of assessment by OSATS form	79.80 ± 18.32
Boxtrainer and tasks	
The box is easy to set up at home	59.00 ± 13.46
The box is valuable for acquisition of laparoscopic skills	93.00 ± 9.38
How well do the tasks test your laparoscopic skills	68.40 ± 12.60*
How useful are the tasks for laparoscopic training?	79.40 ± 15.19*
Vision	
I have other surgical interests/ambitions in surgery than MIS	45.00 ± 30.74
Training at home to develop FLS should be mandatory	83.17 ± 18.21
Training should be mandatory before practicing laparoscopy at the OR	76.17 ± 18.44
I prefer training in a skills lab	27.33 ± 19.08
My skills are improved	95.17 ± 7.11
My self-confidence considering performing laparoscopic surgery is improved	92.83 ± 9.20

**Table 7:** Face validity (box trainer)

Boxtrainer	
Design/size	4.00 ± 0.63
Screen/visualization	4.33 ± 0.82
Light source	4.00 ± 0.63
Instruments	4.00 ± 1.26
Tablet software	4.17 ± 0.75
Box mobility	4.17 ± 0.41
User-friendliness	3.33 ± 0.82
Task instructions	4.50 ± 0.55

Presented as mean ± SD (*5 point Likert scale*)

**Table 8:** Face validity (training tasks)

Exercises	Task 1	Task 2	Task 3	Task 4	Task 5	Task 6
Hand-eye coordination	4.67 ± 0.52	4.33 ± 0.52	4.50 ± 0.55	3.50 ± 0.84	4.17 ± 0.75	4.83 ± 0.41
Depth perception	4.67 ± 0.52	4.00 ± 1.26	4.17 ± 0.75	3.50 ± 0.84	3.83 ± 0.98	4.83 ± 0.41
Inverse movement (*fulcrum effect*)	4.17 ± 0.41	3.50 ± 0.84	4.67 ± 0.82	3.50 ± 0.84	4.33 ± 0.82	4.67 ± 0.52
Bimanual dexterity	4.50 ± 0.55	4.00 ± 0.89	4.83 ± 0.41	Inapplicable	4.17 ± 0.75	4.67 ± 0.52
Complementary use of both hands	4.33 ± 0.82	4.17 ± 0.75	4.67 ± 0.52	Inapplicable	4.17 ± 0.98	4.67 ± 0.52
In general	4.50 ± 0.55	4.33 ± 0.82	4.50 ± 0.55	4.00 ± 1.15	4.17 ± 0.75	4.67 ± 0.52

Presented as mean ± SD (*5 point Likert scale*)

First limitation is the small number of participating residents. Although we found a reduction of all mean parameters outcomes, the large population variation prevented significant outcomes when the pre- and post-course data were compared. We would recommend to iterate in executing this research protocol with a larger set of subjects. A power analysis for this study indicated a number of 13 participants will result in significance in all parameter outcomes comparisons [20].

Another limitation were working hour schedules. Assessment dates were depended on resident’s and trainer’s OR schedules. Therefore, the six residents in this study trained for approximately 3 weeks, but there were differences in exact timespan, which may effected our outcomes. Besides that, trainees were asked to train for a minimum of 15 min per training, for 4 or 5 times per week to insure enough data for analysis. The rest of the training protocol was left for interpretation by the residents, so the amount of time spend on at home training differed.

The ForceSense system’s performance outcomes represents tissue manipulation and therefore tissue handling safety. Besides improving on task completion time and instrument handling efficiency, residents can now train themselves to reduce the amount of force exerted on tissue in a home training setting. As for this study, only standard existing parameters were used; it is advisable to tailor motion, force, and time data, into stronger representing new parameters that better fit the OSATS questions. Based on previous research, we expect this would lead to decreased risk and complication for MIS and improved patient safety in the operating room [17, 26]. Further research is needed to determine the effect on costs and cost-effectiveness. A decrease in costs for laparoscopic training and MIS in operating room is expected, considering the steep learning curves and less demand for direct supervision by senior surgeons.

After statistical analysis, we concluded that ForceSense outcomes representative for tissue manipulation and instrument handling decrease after sufficient amount of training at home. Proficiency levels were reached for each task within a reasonable period of time. Therefore, we can conclude that fundamental laparoscopic skills are acquired and this course is effective for training in MIS. We concluded that the acquired skills were dependent on the type of tasks offered. This information can be considered valuable for course directors and trainers.

## Appendix

Description of training tasks as used by Schreuder et al. [13].

Due to practical consideration, task 3 “Pea on a peg” in the original protocol is replaced by “Flap task”.

**Training task 1**: “Post and sleeve”: The six sleeves are positioned on the left side of the board. The sleeves have to be picked up with the left hand, passed over to the right hand and then transferred to their mirrored posts on the opposite side of the board. After the six sleeves have been moved successfully to the other side, the training task is to be repeated in the opposite direction, now starting with the right hand.

**Training task 2**: “Loops and wire”: The board is positioned with four loops in front, two pipe cleaners are lying in front. The first pipe cleaner has to be introduced through the first row of loops with the right instrument, from the right side of the board. After passing the rings in the first row, the pipe cleaner should be taken with the left instrument. After successfully moving the pipe cleaner through all rings, the training task is repeated with the second pipe cleaner, the opposite direction, through the second row of rings.

**Training task 3**: “Flap task”: The flap attachment is positioned on the left side of the board. The wire is positioned on the flap. The flap must be lifted and the wire must be inserted in the front hole of the flap from above. The flap must be turned over and the wire should be inserted in the back hole of the flap from underneath. The task is finished when the wire is all the way through and the knot is resting on the front hole.

**Training task 4**: “Wire chaser (one hand)”: The board is positioned with the text ‘one hand’ in front. Three rings, with decreasing diameter, must be transferred one-by-one to the other side of the wire, using the dominant hand.

**Training task 5**: “Wire chaser (two hands)”: The board is positioned with the text ‘two hands’ in front. Three rings, with decreasing diameter, must be transferred one-by-one to the other side of the wire, starting with the dominant hand. Both hands are used and hands need to change after each curve in the ring.

**Training task 6**: “Zigzag loops”: The board is positioned with four loops in front, the rope is lying in front. The rope must be passed through the four loops of the first and second row of the loop-board, resulting in a zigzag pattern. This training task has to be performed using both hands, starting from the right side.
